# The Immunology of type 1 diabetes

**DOI:** 10.1038/s41577-023-00985-4

**Published:** 2024-02-02

**Authors:** Kevan C Herold, Thomas Delong, Ana Luisa Perdigoto, Noah Biru, Todd M. Brusko, Lucy S.K. Walker

**Affiliations:** 1 Departments of Immunobiology, Yale University, New Haven, CT; 2 Departments of Internal Medicine, Yale University, New Haven, CT; 3 Anschutz Medical Campus, University of Colorado, Denver, CO; 4 Internal Medicine, VA Connecticut Healthcare System, West Haven, CT; 5 Department of Pathology, Immunology and Laboratory Medicine, University of Florida Diabetes Institute, Gainesville, FL, USA; 6 Institute of Immunity & Transplantation, University College London, Division of Infection & Immunity, London, UK

## Abstract

Following the seminal discovery of insulin a century ago, treatment of individuals with type 1 diabetes (T1D) has been largely restricted to efforts to monitor and treat metabolic glucose dysregulation. The recent regulatory approval of the first immunotherapy that targets T cells as a means to delay the autoimmune destruction of pancreatic β-cells highlights the critical role of the immune system in disease pathogenesis and tends to pave the way for other immune-targeted interventions for T1D. Improving the efficacy of such interventions across the natural history of the disease will probably require a more detailed understanding of the immunobiology of T1D, as well as technologies to monitor residual β-cell mass and function. Here we provide an overview of the immune mechanisms that underpin the pathogenesis of T1D, with a particular emphasis on T cells.

## Introduction

Type 1 diabetes (T1D) is an autoimmune disease that results in the killing of pancreatic islet β-cells, leading to metabolic failure requiring lifelong insulin treatment. T1D occurs in individuals with a genetic predisposition, in whom disease onset and progression is triggered by environmental or immunological events. Although the need for multiple factors — genetic, environmental and immunological — to induce the disease would suggest that T1D is a rare event, it is not uncommon. In 2021, there were an estimated 8.4 million people worldwide with T1D, with around 510,000 new cases diagnosed in that year^1^. Of these, 18% were younger than 20 years, and 64% were aged 20–59 years. The remainder were aged 60 years or older. Approximately 35,000 non-diagnosed individuals died within 12 months of symptom onset. The rates between countries vary greatly: 20% of individuals with T1D were in low-income and lower-middle-income countries. With modelling, using random-forest regression of published T1D mortality data, the remaining life expectancy for a 10-year-old individual diagnosed in a high-income country was reduced by approximately 11 years and in a low-income country by 49 years (ref. 1).

T1D develops in a clinically silent manner over a period of months to years. Its progression has been defined by key parameters that appear after the initiation of autoimmunity. Stage 1 is defined by the presence of two or more autoantibodies, stage 2 is defined by two or more autoantibodies and β-cell dysfunction as β-cell mass is progressively eliminated, and stage 3 equates to clinical T1D. Individuals diagnosed as stage 1 have a 35–50% risk of progressing to clinical T1D within 5–6 years. This risk is raised to 75% for those at stage 2, with a median time to diagnosis of 2 years^2,3^ (Table 1).

Here we review the immunological basis of T1D and discuss how preclinical and clinical studies, as well as investigations of human samples, have provided new insights and opportunities for the development of therapeutics. We also provide guidance for interventions that prevent disease development or induce remission of the disease.

## Genetic factors in T1D

Relatives of individuals with T1D have a significantly increased risk of developing the disease, but almost 90% of newly diagnosed individuals do not have a relative with the disease^4^. In first-degree relatives, the risk ranges from 3% in children of female probands to 5% in children of male probands, 8% in siblings and can be greater than 70% in identical twins^5–7^. The human leucocyte antigen (HLA) genes account for approximately 40–50% of familial predisposition^8^. The most frequent HLA haplotypes associated with T1D are DR3-DQ2 and DR4-DQ8, which are present in up to 90% of individuals with T1D (refs. 5,8). The highest-risk HLA genotype (DR3/DR4-DQB1*03:02) has an odds ratio of greater than 16 for the diagnosis of T1D by the age 15 years (ref. 9). A molecular basis for this genetic association was the finding that protective HLA-DQ alleles encoded an aspartic acid at position 57 of the HLA β-chain, allowing the binding of negatively charged peptide residues^10–12^. The high-risk HLA–peptide complex is speculated to promote the activation of autoreactive diabetogenic T cells.

**Fig. 1:**
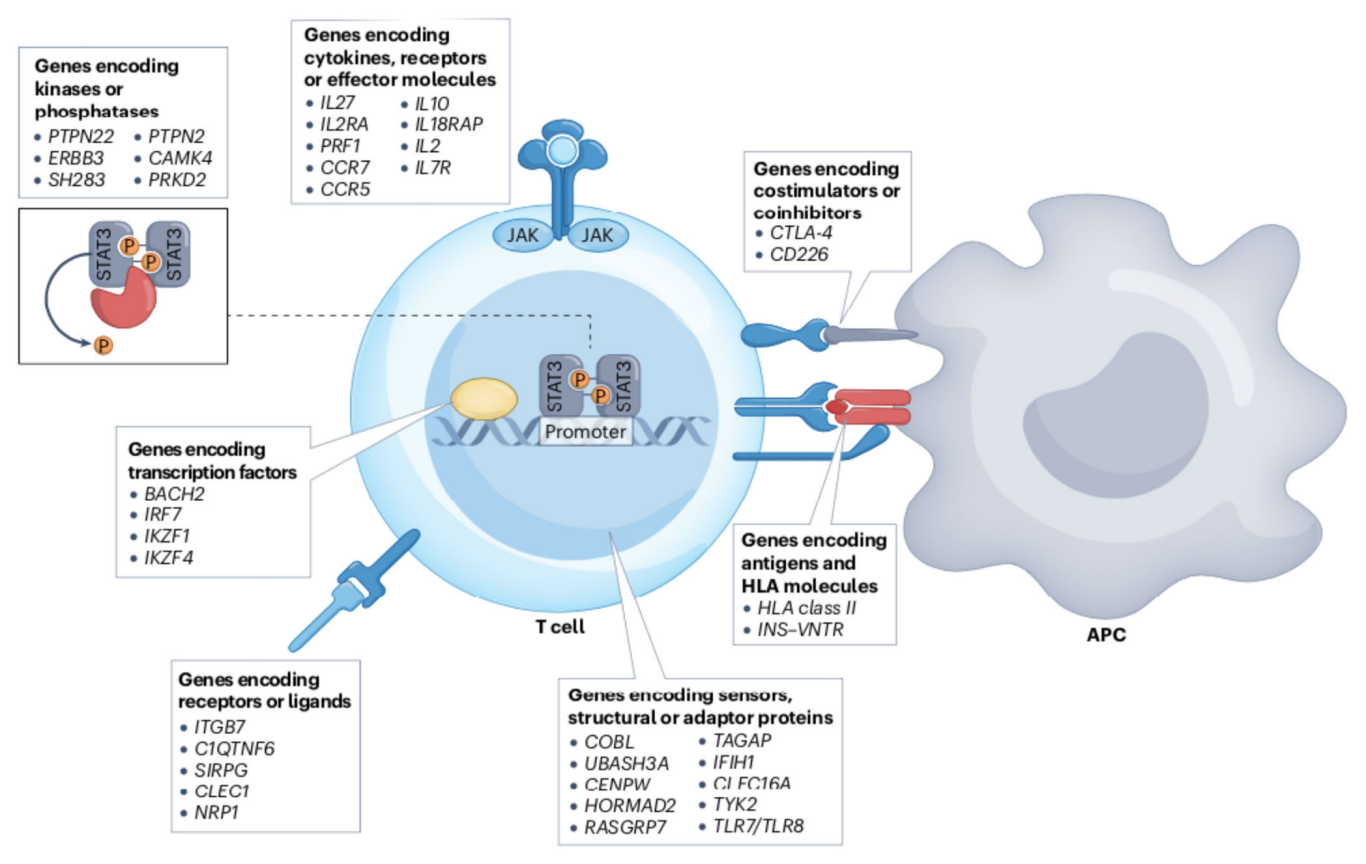
Risk genes for type 1 diabetes encode proteins that impact T cell development and function. Certain haplotypes of the human HLA class II locus and a high number of repeats of the insulin variable number of tandem repeat (VNTR) region have been linked with autoimmune responses in pancreatic islets in individuals with type 1 diabetes. Approximately 50 other candidate risk genes have been found to encode a variety of proteins that are involved in T cell function, activation and differentiation, including kinases and phosphatases, transcription factors, receptors and ligands, cytokines, cytokine receptors and T cell effector molecules, structural and adaptor proteins, and costimulatory or co-inhibitory proteins. Additional risk genes are thought to encode proteins that are involved in T cell activation or differentiation indirectly by modifying antigen-presenting cells or by targeting β-cells (not shown). APC, antigen-presenting cell.

Genetic studies have identified 143 regions of the genome that are associated with susceptibility for T1D, comprising nearly 60 independent candidate genes^13^ (Fig. 1). Substantial genetic insights into the disease have been gained from studies in non-obese diabetic (NOD) mice, which spontaneously develop T1D, for example via comparisons with congenic animals that carry discrete regions of DNA from another strain. Many of the risk loci in both mice and humans are associated with genes that are involved in the regulation of the immune response, including *PTPN22, CTLA4* and *IL2RA* (ref. 5). Reduced expression of IL-2RA has been postulated to account for the reduced fitness and function of regulatory T (T_reg_) cells that is observed in T1D (ref. 14). T1D-associated gene variants are enriched in the open chromatin of T and B cells, and particularly in stimulated CD4^+^ effector T cells, suggesting that they may exert functional effects in these cell types^13^. The second strongest genetic association (after the HLA–MHC haplo- type) involves a variable number of tandem repeats (VNTRs) located upstream of the gene encoding proinsulin (*INS*)^15^. Here, a high number of VNTRs is associated with a high expression of proinsulin in the thymus, which confers a dominant protective effect, purportedly by enhancing T cell tolerance^15–17^.

## Enviromental factors implicated in T1D

A number of large observational studies (ENDIA, GPPAD, TEDDY, DAISY) are currently underway to explore environmental factors such as viral exposure, dietary intake and the microbiome, which have been implicated in T1D development^18–21^. Viral infections have long been suspected to have the potential to break immunological tolerance to self-antigens^22^. The idea that a viral agent can promote a loss in immune tolerance and initiate T1D extends back to the earliest reports of cellular infiltrates in the pancreas of organ donors with T1D (ref. 23). These efforts have been reinvigorated by using modern molecular sequencing for viral identification and histopathological analyses of the pancreas from organ donors with T1D (refs. 24,25). Multiple approaches, including the analysis of stool samples, established a link between enterovirus infection and T1D, and persistent infection is associated with the appearance of islet autoantibodies and progression to overt clinical disease^26^. Establishing causal relationships between T1D and specific viruses remains challenging, as it would require intensive sampling of large populations over long periods of time to capture the timing of infection relative to the initiation of islet autoimmunity and T1D. Furthermore, enterovirus infection might be just one of many possible viral triggers for T1D, adding to the challenge of unravelling causality^27^. An intriguing recent observation concerns the efficacy of the antivirals pleconaril and ribavirin in new-onset T1D in a phase II randomized trial^28^. It has also been suggested that the incidence of T1D increased during the COVID-19 pandemic in certain populations^29,30^. However, a direct association between SARS-CoV-2 infection and islet autoimmunity has not been established^31,32^.

**Fig. 2:**
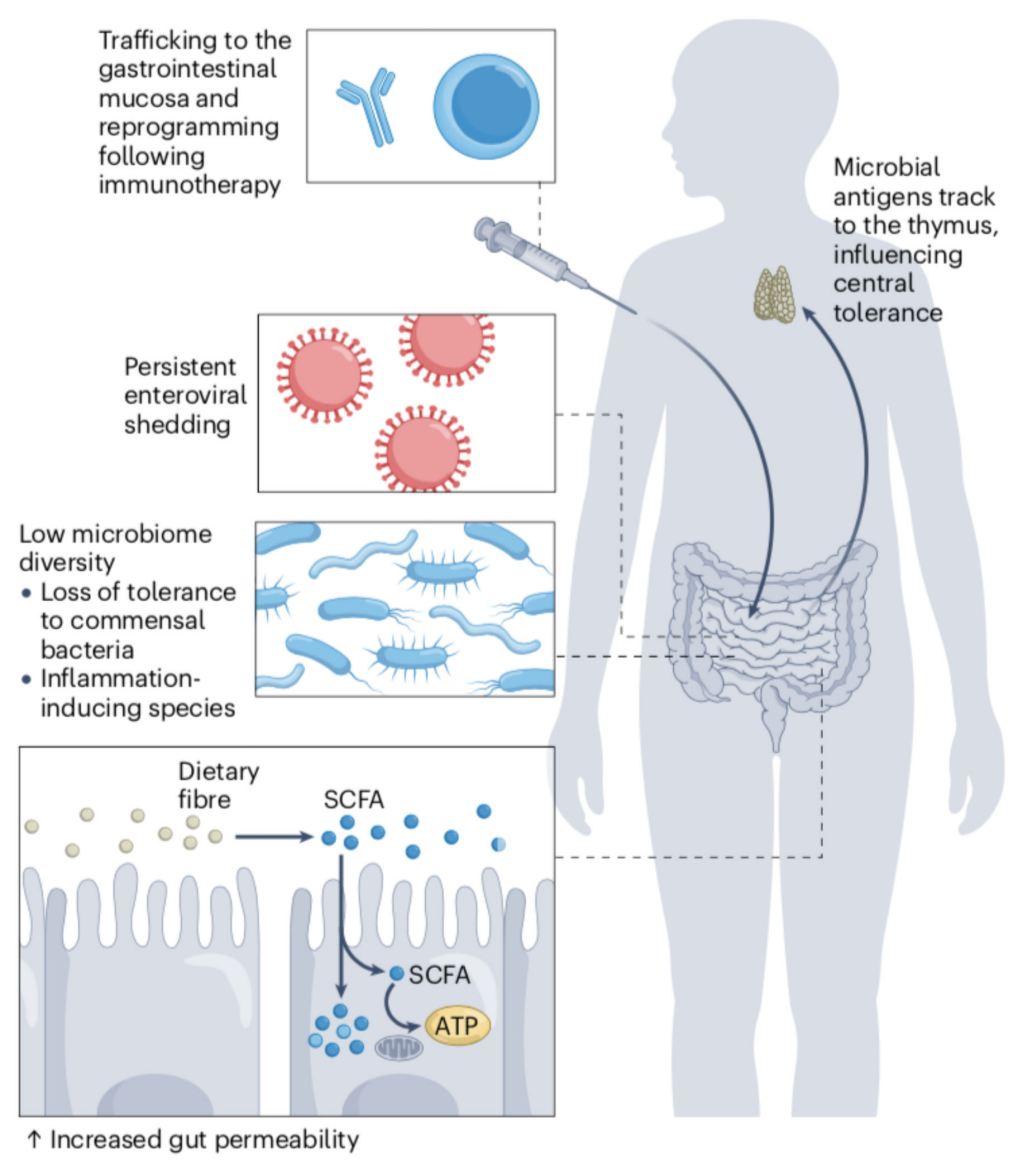
The influence of the microbiome in T1D. Several mechanisms have been proposed to explain associations of the microbiome with type 1 diabetes (T1D). An analysis of stool samples established a link between enterovirus infection and T1D, and persistent infection is associated with islet autoimmunity and progression to overt clinical disease^26^. Invariant immune cells such as mucosal-associated invariant T cells, innate lymphoid cells and others have a role in the maintenance of the intestinal barrier. Their dysfunction and loss of microbiome diversity can lead to loss of tolerance to commensal bacteria and allow the outgrowth of inflammation-inducing species that may affect local tolerance mechanisms, enhance the function of autoreactive cells or even activate immune cells that have cross-reactivity between commensal bacteria and autoantigens^36^. In addition, metabolic products of the microbiome, such as short-chain fatty acids (SCFA), may affect systemic immune regulation, including via direct effects on mucosal-associated invariant T (MAIT) cells^34,37,38,229,230^. Antibodies to commensal microbiota have been identified in individuals at risk for T1D, and in non-obese diabetic mice, cross-reactive antigens that are recognized by CD8^+^ T cells were found (not shown)^24,25,117^. Moreover, microbial exposure may also affect the efficacy of immunotherapies for T1D (ref. 39).

Individuals with T1D often have features of a dysregulated microbiome, possibly linked to changes in gut permeability^33^ or viral infection^34,35^. Interestingly, high-risk HLA haplotypes (such as HLA class II DR3/DR4-DQ2/8) were associated with a potential loss of tolerance and antibody development to host commensal microorganisms^36^. The microbiome also affects mucosal invariant T cells, innate-like T cells that function to preserve gut-barrier integrity, which have also been implicated in the pathogenesis of T1D (refs. 37,38). The ability of the microbiome to alter multiple aspects of immunity suggests that it tends to affect not only the initiation and progression of T1D but potentially also responses to immune modulating therapies^39^ (Fig. 2).

## The role of innate immunity

Innate immune cells, such as dendritic cells, macrophages, neutrophils and natural killer (NK) cells, are thought to play a role at the earliest stages of T1D. Dendritic cells in the pancreatic lymph nodes serve as antigen-presenting cells that can secrete inflammatory mediators such as IL-12 and IL-15, which can promote the expression of costimulatory molecules and activate autoreactive T cells^40–42^. Macrophages that reside in pancreatic islets have also been implicated in both the initiation and the ongoing destruction of pancreatic β-cells, in part through their ability to secrete cytokines (such as TNF and IL-1β, which can stimulate the secretion of IL-6) and reactive oxygen species^40,43–48^. In NOD mice that have received pathological (BDC2.5) CD4^+^ T cells or have been treated with monoclonal antibodies (mAbs) targeted at PD1 to accelerate disease onset, the depletion of macrophages can prevent the disease^49,50^.

Neutrophils are present in the exocrine pancreas and may promote the initiation of T1D by secreting cytokines and chemotactic factors that impact other immune cells, including macrophages and dendritic cells ^40,51–57^. The role of NK cells in T1D is not fully understood, perhaps in part owing to the diversity of NK cell types, but they have also been linked to T1D pathogenesis^58,59^.

Inflammatory mediators such as IL-1 and type 1 interferons are involved in the innate immune response in T1D. Patients with recently diagnosed T1D have higher serum levels of macrophage-derived IL-1β compared with controls, and monocytes from individuals with T1D secrete elevated levels of IL-1β and IL-6 (refs. 60,61). Moreover, NOD mice and humans with T1D have higher expression of interferon (IFN) response markers in islet cells, and a transient IFN-induced gene signature precedes the emergence of islet autoantibodies in humans with T1D (ref. 62). Blocking the IFNα receptor in young NOD mice delayed onset of diabetes, pointing to a role for this cytokine early in the disease process^63–66^. Mechanistically, IFNα has been shown to promote the presentation of self-antigens by islet cells and, consequently, the detection of these cells by cytotoxic T cells. Moreover, it induces the secretion of various chemokines that are involved in the recruitment of immune cells such as T cells and NK cells^62,67^. For example, pancreatic islets from individuals with T1D were shown to express IFN-responsive genes that are involved in antigen presentation, endoplasmic reticulum stress and apoptosis^68–71^. IFNα can also act in synergy with IL-1β to induce β-cell killing^68–71^. A polymorphism in the *TYK2* gene, which encodes a Janus kinase (JAK) that is responsive to IFNα, is associated with T1D, and knocking out *TYK2* in human stem cell-derived islets reduces their sensitivity to T cell-mediated killing^72^.

## Autoantibodies and B cells

The likelihood of progression to T1D can be predicted by measuring islet autoantibody levels and specificities^73^. In individuals with a genetic predisposition to T1D, the first autoantibodies generally appear at ages 1–2 years and are specific for insulin, or at ages 4–5 years and are specific for either glutamic acid decarboxylase (GAD) or insulin^74^. Genetic factors may dictate whether GAD-binding (typically in individuals carrying HLA-DR3) or insulin-binding (typically in individuals carrying HLA-DR4) autoantibodies appear first. In an analysis of 24,662 participants from five prospective studies, the progression of autoantibody reactivity was shown to differ by age, sex and HLA-DR haplotype^75^. Currently, a model that combines a genetic risk score with the number of islet autoantibodies provides the best prediction of progression to T1D (ref. 76). The trigger that initiates the appearance of the first autoantibodies remains elusive but could conceivably encompass β-cell damage or stress, possibly secondary to viral infection(s). Intriguingly, a small increase in postprandial blood glucose can be detected around 2 months before seroconversion, supporting the idea that β-cell perturbations may be associated with the initiation of autoantibody production^77^.

Autoantibodies in T1D are not believed to be pathogenic. However, B cells have an important role in disease pathogenesis, most probably as antigen-presenting cells. Loss of B cell antigen presentation, but not loss of antibody secretion, is sufficient to prevent the disease in the NOD model^78,79^. NOD mice with B cells specific for the model antigen HEL, which were unable to take up islet antigen through their B cell receptor, were protected from T1D development and lacked T cell responses to the islet autoantigen GAD^80^. Conversely, selective B cell recognition of insulin can enhance diabetes in NOD mice^81^. Loss of anergy of high-affinity insulin-binding B cells has been identified in patients prior to and at the time of T1D diagnosis ^82^. Interestingly, people diagnosed with T1D at <7 years of age have a higher number of B cells in the islet infiltrate and fewer remaining β-cells than those diagnosed at an older age ^83^.

## Features of autoreactive T cells

**Fig. 3:**
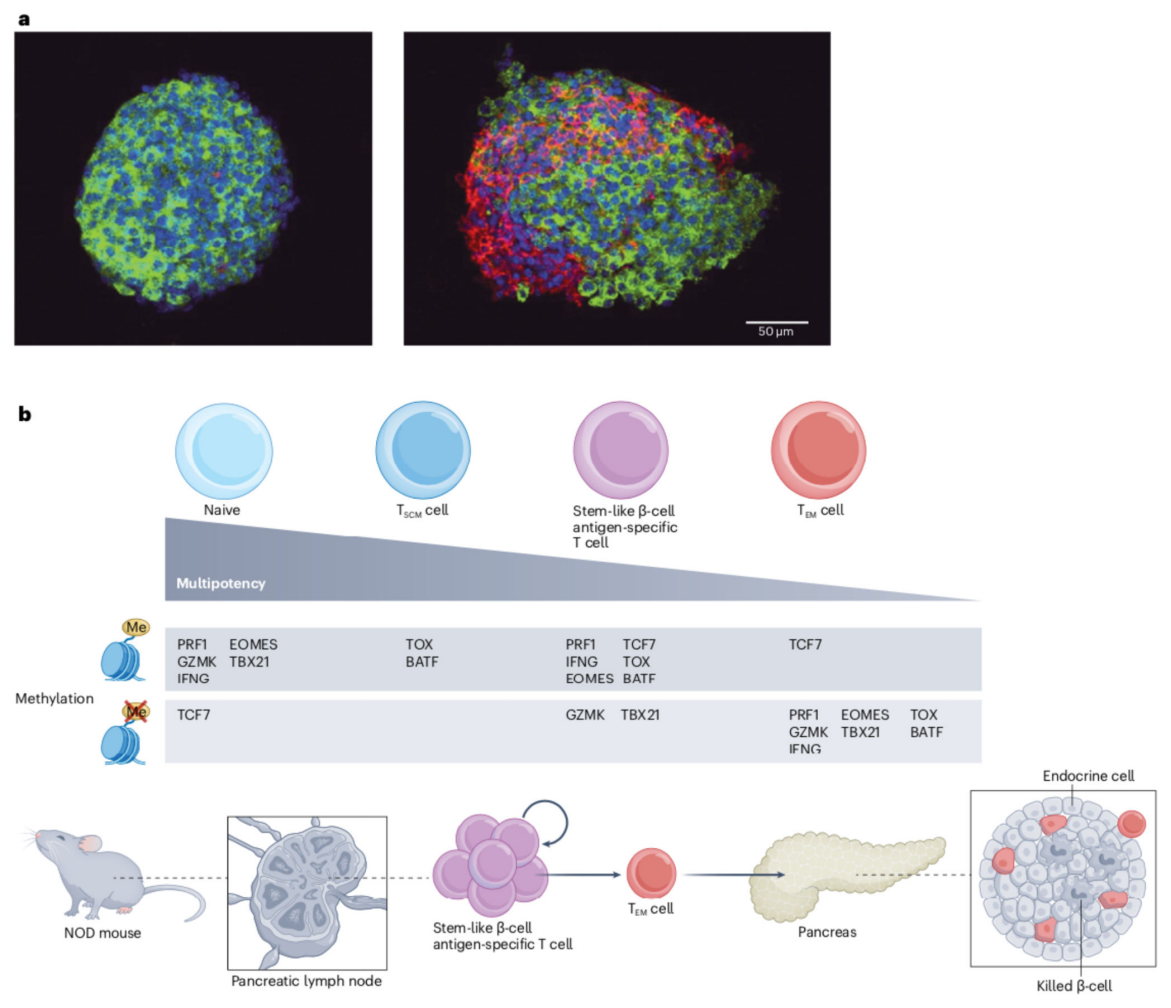
Insulitis and CD8^+^ T cells in type 1 diabetes. **a**, Confocal microscopy of islets from a 12-week-old non-obese diabetic (NOD) mouse. Islets were stained for insulin (green), CD45 (red) and nuclei (blue). The two examples shown are of an islet without insulitis (left) and with immune cell infiltration (right) from the same mouse, illustrating the heterogeneity of β-cell destruction during type 1 diabetes pathogenesis. (Image taken in the Yale Center for Cellular & Molecular Imaging Confocal Facility). **b**, Stem-like autoreactive CD8^+^ memory T cells targeting β-cell antigens in type 1 diabetes have properties of both stem memory T (TSCM) cells and effector memory T (TEM) cells. Middle row: the stem-like population has a genome methylation profile that reflects this intermediate phenotype, which is reflected by the methylation patterns of key transcription factors related to differentiation (EOMES, TBX21, TCF7), effector functions (PRF1, GZMK, IFNγ) and exhaustion (TOX, BATF). Bottom row: in NOD mice, this stem-like population of T cells resides in the pancreatic lymph nodes, wherein the cells can differentiate into short-lived TEM cells that migrate to the pancreas^115^ and destroy β-cells.

An abundance of data from NOD mice and other preclinical models indicates an important role for T cells in T1D development and progression. Evidence for a pathogenic role of T cells in humans was provided by clinical studies four decades ago, when it was shown that treatment with cyclosporin A, an immunosuppressive drug that suppressed T cell receptor signalling, promoted the preservation of β-cell function (determined by measuring stimulated C-peptide levels, a by-product of insulin production) and reduced requirements for exogenous insulin. Cyclosporin A or chronic broad-spectrum immune-suppressive drugs (such as a combination of azathioprine with prednisone) were abandoned largely because of toxicity concerns. Information about the architecture, participating cells and mediators in autoimmune insulitis has been greatly expanded with the availability of pancreases from deceased donors obtained by the Network for Pancreatic Organ donors with Diabetes programme (nPOD; www.jdrfnpod.org), as well as from pancreatic biopsies from living individuals with T1D (DiViD; www.oslodiabetes.no)^84^. Tissue imaging mass cytometric analysis of human T1D pancreatic tissue revealed that CD4^+^ and CD8^+^ T cells are recruited to the islets simultaneously, with a trend for insulitis to be associated with islets that retain insulin-containing β-cells^85^. In human insulitis, CD8^+^ T cells outnumber CD4^+^ T cells^86,87^. Of note, in both NOD mice and human insulitis, not all islets are affected equally: within the same pancreas and even within the same region, there may be islets that are completely free of immune cells and others that show more substantial cellular infiltrates (Fig. 3a). The reason(s) for this heterogeneity remain unclear. The extent of insulitis is inversely correlated with the duration of diabetes, with one study identifying insulitis in all donors examined within 1 year of diagnosis and in 19% of those with disease durations of longer than 1 year^87^.

### CD4^+^ T cells

CD4^+^ T cells specific to proinsulin, GAD and insulinoma-associated protein-2 (IA2) have been isolated from the pancreatic islets of organ donors who had T1D (refs. 88–90). In NOD mice, many of the islet-infiltrating CD4^+^ T cells bind a fragment of the insulin B chain (amino acids B:9–23), and a single amino acid mutation in this region abolishes immunogenicity and the disease^91^. Autoantigen-reactive CD4^+^ T cells can also be identified in the peripheral blood using tetramer staining or peptide restimulation coupled with activation marker expression. The frequencies of activated (CD38^+^) islet antigen-specific memory CD4^+^ T cells (reactive against GAD65, IGRP, ZnT8, ChgA, PPI_78-90_^K88S^ or PPI_35-47_^R46E^) in the peripheral blood are elevated in people with T1D (ref. 92) (Table 1). Pathogenic CD4^+^ T cells in T1D were initially thought to have a T helper 1 (T_H_1) phenotype, with T_H_2 cytokines generally regarded as protective; however, in recent years, this view has become more nuanced^93^. Some evidence suggests a pathogenic role for a T_H_17 response in T1D (refs. 94,95).

**Fig. 4:**
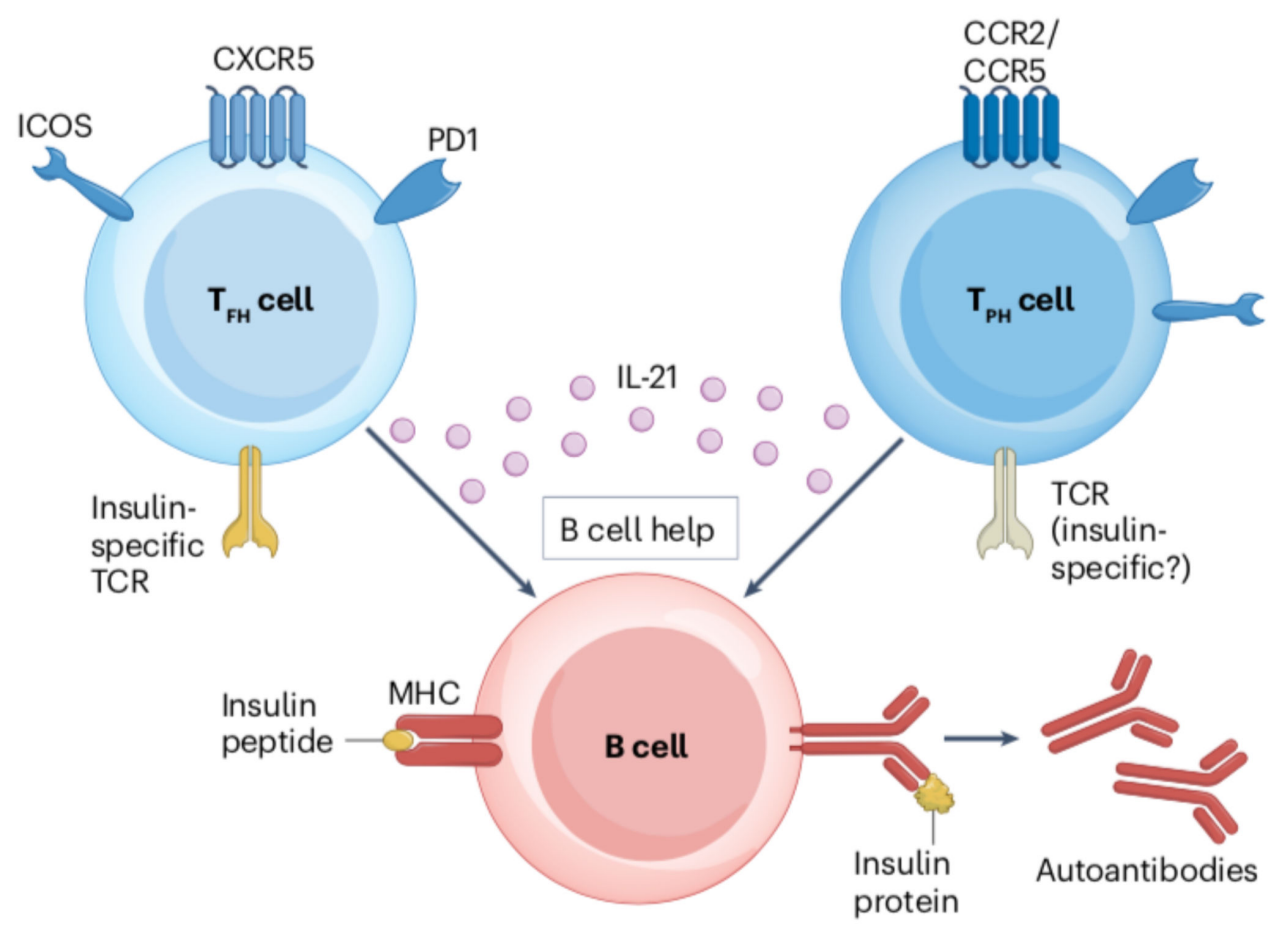
T cell phenotypes associated with B cell help are linked to T1D. Elevated levels of T cells with a follicular helper (T_FH_) phenotype and increased IL-21 production by T cells have been noted in mouse models and individuals with T1D (ref. 101). In mouse models of diabetes, insulin-specific T cells can exhibit a TFH phenotype and provide help to B cells that mount insulin-specific antibody responses^231^. Insulin-specific T cells with a TFH phenotype have also been documented in individuals who have recently developed islet-targeted autoantibodies^232^. T cells with a T peripheral helper (T_PH_) phenotype are also increased in individuals at onset of T1D and in at-risk individuals who go on to develop diabetes^100^. Like TFH cells, TPH cells expresses inducible T cell costimulator (ICOS) and PD1 but lack expression of CXCR5 and instead can express chemokine receptors associated with migration to inflammatory sites (for example, CCR2 and CCR5). Whether TPH cells recognizes T1D-associated antigens such as insulin remains unclear. TCR, T cell receptor.

Intriguingly, an increased frequency of circulating T cells with a follicular helper (T_FH_) phenotype has been noted in individuals with T1D, and the hallmark T_FH_ cytokine IL-21 appears to be elevated, consistent with a role for T_FH_ cells in pathogenesis^96–98^ (Fig. 4). T_FH_ cells support the formation of ectopic B cell germinal centres. A recent work suggests that ectopic germinal centres form in the thymus of NOD mice, supported by local T_FH_ cells^99^. T peripheral helper (T_PH_) cells, which are phenotypically similar to T_FH_ cells but thought to act in peripheral tissues, are also increased at T1D onset and in at-risk individuals who subsequently progress to T1D (ref. 100). T_FH_ and T_PH_ cells are highly sensitive to costimulation through the interaction of CD28 with CD80/ CD86 (ref. 101), which means that they may be targeted by drugs that block these receptors. One such drug is abatacept, a fusion protein composed of the extracellular domain of CTLA4 (a CD28 homologue that binds CD80/CD86 with higher affinity) linked to a modified Fc portion of human IgG1.

Both T_FH_ and T_PH_ cells interact with B cells. Tertiary lymphoid organ (TLO)-like structures, which are aggregates of T cells and B cells that can be organized to some extent by tissue stroma^102^, have been identified in the pancreas of NOD mice^103^, but the presence of these structures in human pancreases has been uncertain. Recently, a study of organ donors has shown that out of 21 individuals with T1D who had insulitis, 12 had evidence of pancreatic TLOs. Curiously, the mean age of diagnosis was significantly lower in donors with TLOs than in those without, and well-organized TLOs were only found in individuals with very short disease duration. TLOs were also identified in three out of six autoantibody-positive non-diabetic donors examined^104^.

### CD8^+^ T cells

Autoantigen-reactive CD8^+^ T cells can be identified in the pancreas and peripheral blood of individuals with T1D (Fig. 3b), as well as in the peripheral blood of control participants^105–107^ (Table 1). There is an enrichment of islet autoantigen (such as ZnT8_186–194_)-reactive CD8^+^ T cells in the pancreas of T1D donors in spite of the similar frequency in blood from individuals with T1D versus non-diabetic controls^108^. The proportion of patients with islet-infiltrating preproinsulin (PPI)-reactive CD8^+^ T cells is estimated to be as high as 60–70%, and these cells can kill human β-cells in vitro^109,110^. Interestingly, CD8^+^ T cells with different autoantigen specificities can be found within the same islet, particularly in patients with long-standing disease^111^.

The differentiation state of islet-reactive CD8^+^ T cells in the peripheral blood has been investigated in several studies. Notably, a 2-year longitudinal study in children under 12 years of age with T1D has found that the frequency of CD57^+^ effector memory islet-specific CD8^+^ T cells declined as C-peptide levels fell, consistent with a role for antigen load in promoting the antigen-specific T cell response^112^. An exhaustion-like profile, with expression of EOMES, 2B4, PD1, TIGIT and CD160, was associated with slow disease progression^113^. Compared with responses to conventional antigens such as viral antigens, the interactions of CD8^+^ T cells with autoantigens displays lower avidity but also longer persistence. An analysis of the epigenome of T1D-specific autoantigen-reactive CD8^+^ T cells (identified with class I MHC tetramers) revealed stem-like epigenetic features, which are postulated to account for the longevity of these cells despite constant exposure to autoantigen^114^. Consistent with this, autoantigen-reactive CD8^+^ T cells in the pancreatic draining lymph node in NOD mice exhibited a high self-renewal capacity with the ability to differentiate into short-lived effector cells that can destroy β-cells^115^. Because islet-reactive CD8^+^ T cells are found in healthy individuals^108,113,116^, how these cells are triggered to initiate immune pathology is an open question. Interestingly, commensal bacteria within the gut microbiome can represent a source of antigenic peptides that cross-react with IGRP-specific CD8^+^ T cells, suggesting a mechanism by which the microbiome might modulate the course of the disease^117–119^.

## Crosstalk between β-cells and immune cells

There is evidence that crosstalk between immune cells and β-cells contributes to T1D pathogenesis because β-cells release insulin peptide fragments into the circulation that can stimulate CD4^+^ T cells, even at distant sites^120^. Pro-inflammatory cytokines (such as IFNγ and TNF) can induce the expression of class I and II HLA molecules on β-cells^121^. The hyperexpression of HLA molecules in pancreatic islets of individuals with T1D, which is thought to enhance islet antigen recognition, was first described over 40 years ago^122,123^. At early stages of the disease, this may involve IFNα acting via a TYK2-STAT2-IRF9 axis, and at later stages, IFNγ-driven STAT1 activation may contribute to high levels of HLA expression^69,72,124^.

Dying or senescent β-cells provide a source of antigen for auto-reactive T cells^125^. Inflammatory conditions in the pancreas induce the dioxygenase TET2 in β-cells, and *Tet2*-deficient mouse β-cells are resistant to killing by T cells^126^. Separately, thioredoxin-interacting protein (TXNIP) can mediate oxidative stress, inhibit cell proliferation and induce apoptosis by inhibiting the thioredoxin system. Elevated levels of TXNIP are found in human diabetic islets and induce β-cell apoptosis, whereas β-cell-specific TXNIP deficiency protected against diabetes in NOD and streptozotocin-induced mouse models^127^. The calcium channel blocker verapamil can inhibit TXNIP and was shown to have beneficial effects on β-cell function in individuals with newly diagnosed T1D (ref. 128). Treatment with verapamil reduced the levels of chromogranin, a β-cell autoantigen, and was suggested to decrease the levels of circulating pro-inflammatory T_FH_ cells^129^.

**Fig. 5:**
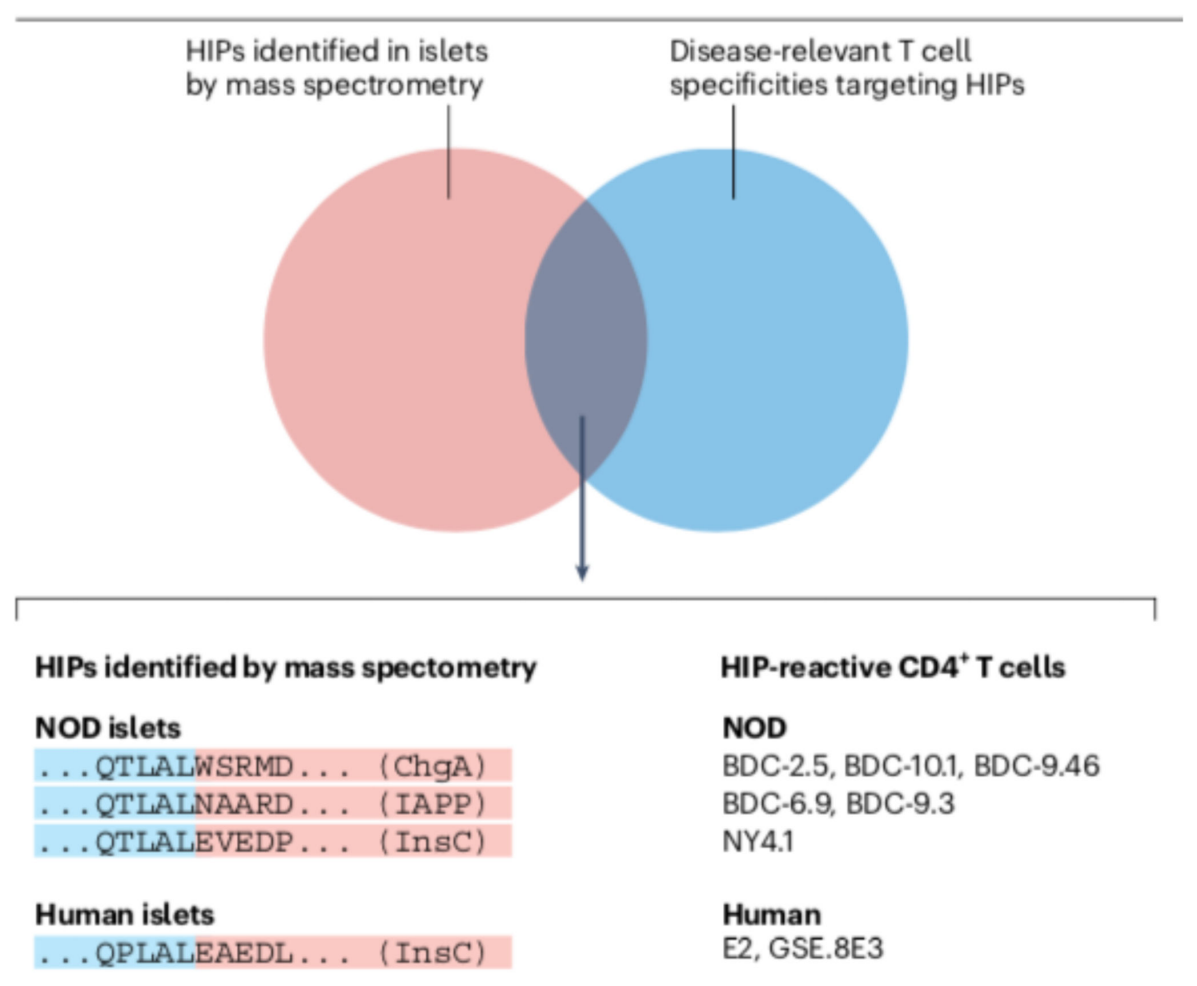
Disease-relevant hybrid insulin peptides (HIPs). The figure shows HIPs that were confidently identified from pancreatic islets by mass spectrometry and for which disease-relevant HIP-specific T cells were also identified in peripheral blood mononuclear cells. For example, the CD4^+^ T cell clone E2 was isolated from peripheral blood mononuclear cells of individuals with recent-onset T1D (ref. 136) or from residual islets of organ donors with T1D (such as T cells expressing the TCR GSE.8E3). Moreover, experiments in NOD mice identified various diabetes-triggering CD4^+^ T cell clones that target HIPs bearing a distinct C-peptide (InsC) fragment (ending in the amino acid sequence LAL) linked to the N-termini of peptides derived from chromogranin A (ChgA), proinsulin (C-peptide) or islet amyloid polypeptide (IAPP). These HIPs are formed by the aspartic protease cathepsin D through a reversed proteolytic transpeptidation reaction. Peptide sequences highlighted in blue originate from C-peptide, whereas sequences highlighted in red originate from ChgA, IAPP or InsC.

Islet proteins may undergo various types of modifications, leading to the emergence of neoantigens that can be recognized by T cells^130,131^. For example, CD4^+^ T cells from a patient with T1D were found to be reactive to an oxidized epitope of the insulin A-chain containing a novel disulfide bridge^132^. Another type of post-translational modification that occurs in β-cells is represented by hybrid insulin peptides (HIPs), which are proinsulin fragments fused to other β-cell peptides through peptide bonds (Fig. 5). The presence of HIPs in human and mouse pancreatic islets has been validated through mass spectrometric analyses^120,133–135^. HIPs differ from conventionally modified epitopes in that they encompass entire amino acid sequences not found in the genome. This results in the introduction of fresh contact residues for interactions with both MHC and T cell receptors (TCRs). Significantly elevated levels of pro-inflammatory (IFNγ-secreting) T cells targeting HIPs have been detected in the peripheral blood of individuals with recent-onset T1D, setting them apart from non-diabetic control subjects^136,137^. HIP-reactive T cells have also been detected in residual pancreatic islets of organ donors with T1D (refs. 88,135,136). Furthermore, various diabetes-triggering CD4^+^ T cell clones that target HIPs have been identified in NOD mice^138–140^. In experiments using NOD mice engineered to possess only a monoclonal population of T cells, it was revealed that only a small subset of 17 autoantigen-specific TCRs tested had the capability to initiate islet infiltration and β-cell destruction independently^141^. Notably, pathogenic TCRs were derived from the CD4 T cell clones BDC-2.5, NY-4.1 and BDC-6.9, all of which respond to HIPs in which a common C-peptide fragment is linked to different β-cell peptides^135,138,140^. Another pathogenic TCR was derived from BDC-10.1, which targets the same HIP as BDC-2.5 (Fig. 5). Lastly, a TCR responsive to the unmodified insulin B:9–23 epitope was also identified as pathogenic; however, its potency was lower, resulting in diabetes in only 33% of the mice, compared with the 100% incidence for both BDC-2.5 and BDC-10.1, as well as the 71% and 56% observed for NY4.1 and BDC-6.9, respectively. The remaining TCRs which were focused on epitopes derived from GAD65, IA2 and phogrin (IA2-beta) were not independently pathogenic. These findings highlight that autoreactivity does not equate to pathogenicity^141^ and that a distinct group of HIPs (2.5HIP, 6.9HIP and HIP11; see Fig. 5) are targeted by the most pathogenic CD4^+^ T cell clones in NOD mice. Recent data established that these pathogenic HIPs are generated owing to the reversed proteolytic action of aspartic protease cathepsin D (CatD)^142^, suggesting that CatD may serve as a therapeutic target in T1D. Other modifications of insulin that have been identified as T cell epitopes in T1D include deamidations, introduced by tissue transglutaminase 2 and different forms of protein oxidation, induced by reactive oxygen species^143,144^. Further forms of post-translational modifications that are linked to disease pathogenesis in individuals with T1D are citrullination and carbonylation of epitopes derived from proteins other than insulin^88,145,146^. Lastly, defective ribosomal initiation products of proinsulin (DRIPs) have also been suggested as T cell epitopes in T1D (ref. 147).

### Failures of regulation: T_reg_ cells in T1D

Several candidate genes associated with T1D, such as *IL2RA, CTLA4, PTPN2* and *PTPN22*, have important functions in T_reg_ cells. There is not a quantitative deficiency of T_reg_ cells in T1D; however, a number of reports suggest that T_reg_ cells in individuals with T1D may be dysfunctional and memory effector T cells from patients may be resistant to suppression. Impaired IL-2R signalling has been suggested as a mechanism by which the metabolic and functional fitness of T_reg_ cells is compromised in individuals with T1D (ref. 149). Defects in the IL-2R pathway and reduced phosphorylation of STAT5 may lead to insufficient FOXP3 expression in T_reg_ cells^14^. Likewise, the production of IFNγ by T_reg_ cells has been found and interpreted as a measure of T_reg_ cell instability in T1D (ref. 150). Cytokines such as IL-12, IL-23 and IL-21 have also been reported to drive T_reg_ cell lineage instability^150,151^, and there may be a preferential expansion of T_FH_ cells over follicular T_reg_ cells in secondary lymphoid organs^152^.

## Immune modulation as a therapy for T1D

**Fig. 6:**
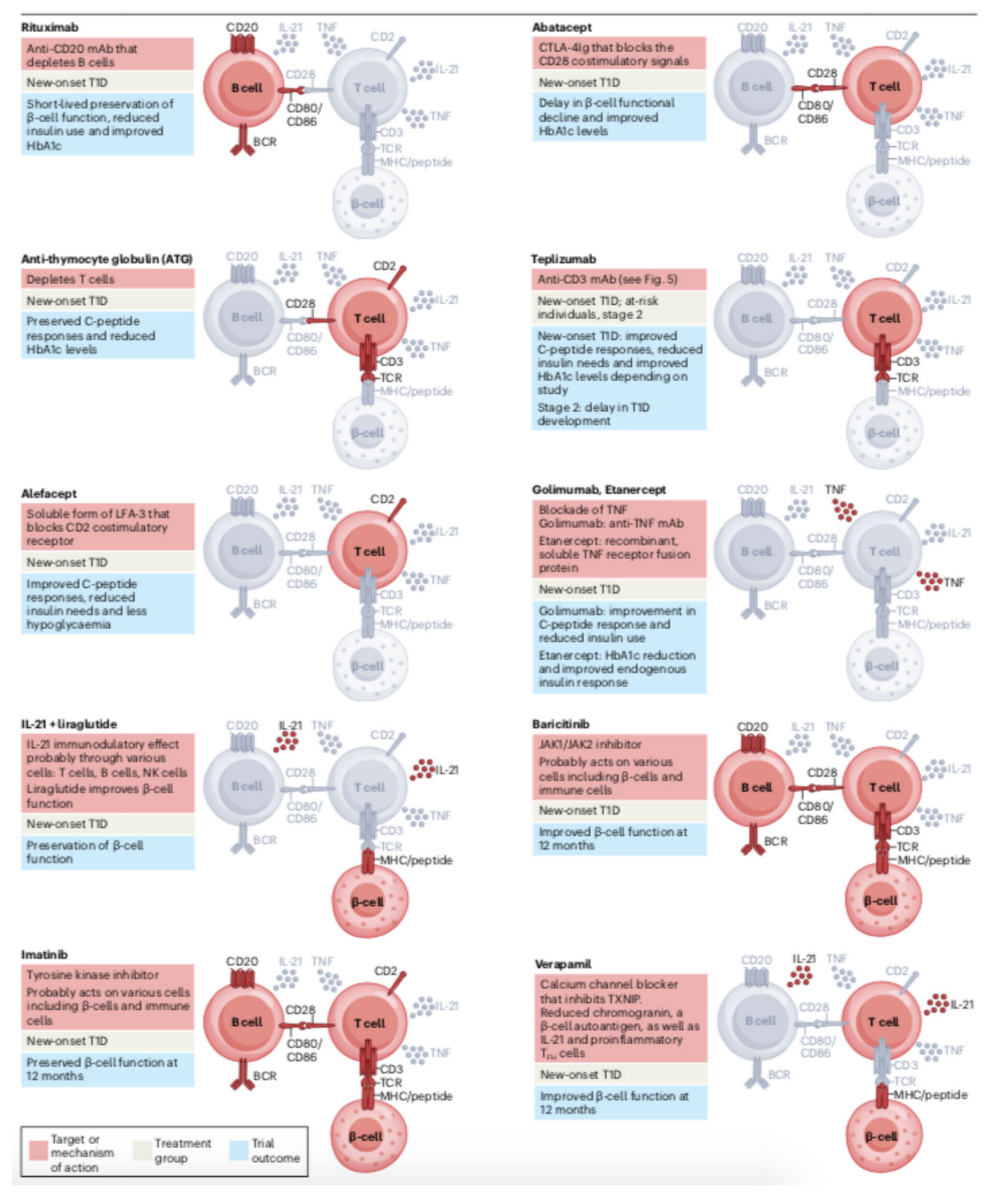
Drugs and mechanisms that have shown efficacy in T1D. The targets of these therapies have included innate and inflammatory mediators and pathways, B cells, costimulatory molecules, T cells and β-cells ^128,158,163,164,173,179,185,188,202,203^. The principle targets of drug action are highlighted in red. The T cells depicted are generic and may include several different subsets (for example, CD4^+^, CD8^+^, TFH cells and others). mAb, monoclonal antibody.

The insights into pathological immune responses in T1D have led to efforts to develop biomarkers of disease activity. Challenges remain because certain parameters that can easily be measured in the serum (such as autoantibodies and cytokines) are not the direct cause of β-cell killing, and the contributions of immune cells measured in the blood may not precisely reflect events in the pancreas. Clinical trials have, therefore, used clinical parameters for defining their entry criteria, but there is heterogeneity in the duration and progression of the disease between participants. This heterogeneity is captured in the idea of disease ‘endotypes’, which are based on immunological and demographic differences between patients and their responses to therapies — age being an important feature^153^. Many studies have enrolled patients with recent-onset stage 3 T1D, most frequently in early teenage years, with a primary end point based on stimulated C-peptide responses compared with placebo control groups (Fig. 6 and Box 1). In the sub-sections that follow, we provide selected highlights from this area and refer the reader to other reviews for more extensive coverage of the field^154–156^.

### Therapies targeting innate immune pathways

Given the early role of innate immune cells in triggering autoimmune diabetes, cytokines and chemokines implicated in innate immune responses have been proposed as potential pharmacological targets. In a clinical study in children with new-onset T1D, etanercept, a fusion protein that binds TNF and blocks its activity, led to a reduction in haemoglobin A1c (HbA1c) and improved endogenous insulin responses^157^. Moreover, a highly successful trial of the TNF-targeting antibody golimumab showed improvement in C-peptide responses and clinical parameters when it was administered to participants aged 6–21 years with stage 3 T1D for ≤52 weeks^158^.

However, trials with agents that block IL-1β (such as the mAbs anakinra or canakinumab) have failed to show benefit^159^. Similarly, tocilizumab, a mAb that blocks IL-6R, did not slow β-cell loss^160^, nor did blocking CXCR1/2, which is important for neutrophil recruitment^54,161,162^. Imatinib, a small-molecule tyrosine kinase inhibitor that affects inflammatory pathways in immune cells and pancreatic β-cells, showed modest effects on C-peptide responses at 1 year^163^.

Because of their pleotropic effects on inflammatory pathways, there is a strong rationale for using JAK inhibitors to treat patients with T1D. Indeed, a pilot trial of baricitinib in patients with new-onset T1D has shown improvement in β-cell function after 1 year^164^.

### Antigens as targets

Antigen-specific strategies such as vaccination have been tested in individuals with T1D and, given their general safety, have also been trialled in patients at risk for stage 3 T1D. Subcutaneous immunization with GAD-alum (Diamyd®), which is thought to modulate T cell responses to GAD65, initially showed improvement in stimulated C-peptide responses, but subsequent trials failed to reproduce this finding^165–167^. It has been suggested that intralymphatic administration of this antigen may be more effective^168^. Parenteral and oral administration of insulin have been trialled to test whether mucosal delivery of antigen affects responses, but these trials have failed to meet their end points^169–171^. In prevention studies, post hoc analyses have suggested that some at-risk individuals who have impaired pancreatic β-cell responses may experience some delay in diagnosis of clinical T1D when treated with oral insulin^171^. A therapeutic dose for orally administered antigens is challenging to identify: doses that are higher than those used in prevention studies have shown greater systemic antigen responses^172^.

### B cell-directed immunotherapy

Given that preclinical data suggest a pathogenic role for B cells in T1D, therapies targeting this cell population have been investigated. A clinical trial testing rituximab, a CD20-targeted mAb that depletes B cells (four doses over 1 month), has been shown to preserve β-cell function and delay disease progression in individuals with new-onset T1D^173^. However, the benefit was short-lived^174^. The initial beneficial effects may be attributable to the ability of the drug to interrupt T cell–B cell collaboration, given that antibody responses to the T cell-dependent antigen phiX174 were blocked. However, the fact that the effects were temporary may be owing to the inability of transient B cell depletion to reset defective early B cell tolerance checkpoints, resulting in repletion with autoreactive B cells^175^. In addition, non-responders had a higher expression of T cell-associated gene expression modules after rituximab treatment, consistent with the idea that T cell responses also need to be controlled during B cell-directed immunotherapy^176^.

### Costimulation blockade

A key control point for adaptive immune responses is the provision of costimulation through the T cell surface receptor CD28. Blocking CD28 costimulatory signals with CTLA-4Ig showed efficacy in NOD mice^177^. A clinical trial of the CTLA-4-Ig molecule abatacept, given at days 1, 14 and 28 and then monthly for 2 years in individuals with recent-onset stage 3 T1D, has shown a reduced decline in C-peptide levels compared with placebo^178,179^. Building on this success, a trial was conducted in patients at an earlier stage of the disease (stage 1), with the idea that earlier blockade of T cell activation would prove more effective. In this trial (TrialNet, TN18), abatacept was given monthly for 1 year, and although it showed a delay in the rate of progression, the result did not meet statistical significance (*P* = 0.11)^180^. In both trials, abatacept reduced the levels of activated T_FH_ cells, but it also reduced the frequency of T_reg_ cells that may have interfered with the efficacy and persistence of the effect^101,181^. The critical role of CD28 for the activation of effectors and regulators suggests that careful choice of timing, and possibly combination therapies (for example, co-administering low-dose IL-2 to preserve T_reg_ cells), may be needed^182^.

### Anti-thymocyte globulin

Broad targeting of T cells was tested with anti-thymocyte globulin (ATG), which is routinely used for the treatment of transplant rejection and consists of rabbit antibodies against human thymocytes. In individuals with T1D it was tested at reduced dosages in order to mitigate the known adverse effects of cytokine release syndrome. A 2-day course of low-dose ATG was shown to preserve C-peptide responses in individuals with established T1D in a pilot trial and in new-onset T1D in a subsequent placebo-controlled trial (TN19)^183–186^. Drug administration led to the depletion of CD4^+^ and CD8^+^ T cells. The way in which ATG causes long-term responses are yet unknown, but may have similarities to the effects of teplizumab on immune cells (discussed in the sub-sections that follow). Specifically, low-dose ATG appears to primarily impact CD4^+^ T cells and promote an exhaustion phenotype, while simultaneously preserving T_reg_ cells^187^.

### Alefacept

Two 12-week courses of the LFA3Ig fusion protein alefacept, which blocks T cell proliferation and activation, reduced loss of stimulated C-peptide responses in new-onset T1D. Importantly, there was also a significant reduction in the frequency of severe hypoglycaemia^188^. It depleted CD4^+^ and CD8^+^ central and effector memory T cells, but preserved T_reg_ cells, thereby increasing the ratios of T_reg_ cells to central and effector memory T cells. These findings supported the strategy of improving the balance between immune effectors and regulators for treatment.

### Teplizumab

Teplizumab, a Fc-receptor non-binding humanized CD3-targeted mAb, was the first immunotherapy for T1D to receive FDA approval (November 2022). The earliest work examining the therapeutic potential of CD3-targeted mAbs began in the transplant field, in which the mAb OKT3 was shown to prevent allograft rejection. However, treatment-related cytokine release syndrome owing to the production of TNF by activated T cells and the development of anti-mouse antibodies limited enthusiasm for its translation in humans^189–191^. Early preclinical experiments in T1D using a modified CD3-targeted mAb consisting of F(ab′)2 fragments of the 145-2C11 mAb have shown that it could prevent the induction of auto-immune diabetes in a mouse model of T1D induced by low-dose streptozotocin, which kills pancreatic β-cells^192^. Another study has found that brief treatment of hyperglycaemic NOD mice with CD3-targeted mAbs (with either the whole antibody or F(ab′)2 fragments) can reverse the disease and, importantly, that the disease did not recur after drug treatment was discontinued^193,194^. This observation highlighted the reversibility of the disease even at a time when hyperglycaemia had set in, which was subsequently shown to entail the recovery of β-cell granulation in cells that were non-functional, but not destroyed, at diagnosis^195^. Two humanized FcR non-binding CD3-targeted mAbs, teplizumab and otelixizumab, were produced and each contains two alanines instead of leucines in the Fc portion of the IgG1 molecule^196^. In a randomized controlled phase II trial of 42 patients, a single 12-day or 14-day course of teplizumab was shown to improve C-peptide responses to a mixed meal even 2 years after treatment, and without the severe cytokine release syndrome that was routinely observed with OKT3 (refs. 197,198). Otelixizumab was tested in a randomized placebo-controlled trial of 80 patients and also showed clinical benefit as reflected by reduced insulin requirements 18 months after the single course of treatment shortly after diagnosis^199^. Importantly, like the preclinical studies and unlike continuous immune therapies in the past, the sustained efficacy did not require continuous administration.

A randomized phase II trial (AbATE), sponsored by the Immune Tolerance Network, tested the effects of two courses of teplizumab, 1 year apart, on β-cell function after 2 years^200^. At the same time, a phase III industry-sponsored trial (Protégé) was initiated, in which teplizumab or placebo was given at varying dosages, at diagnosis and at 6 months, with a second (Protégé Encore) trial initiated afterwards. The Protégé trial used a new composite primary end point of insulin use and HbA1c levels. Despite not meeting the composite end point, β-cell function (as measured by stimulated C-peptide response) was improved with teplizumab compared with placebo, confirming the results from earlier trials. A subsequent study has tested treatment of patients more than 4 months after diagnosis, but in whom clinically significant levels of β-cell function could be detected (defined as a stimulated C-peptide level of at least 0.2 pmol ml^−1^). This trial also confirmed improved effects of the drug on β-cell function, although therapeutic outcomes were not as favourable as those observed in new-onset patients^201^. A combined analysis of data from five studies of teplizumab in patients with stage 3 T1D has shown consistent improvement in stimulated C-peptide responses and reduced insulin usage^202^. Furthermore, a recently completed phase III clinical trial (PROTECT) of children and adolescents with new-onset T1D has confirmed these earlier findings on C-peptide preservation at 18 months after enrolment and suggested improvements in insulin use, continuous glucose metrics, hypoglycaemia and patient reported outcomes^203^.

**Fig. 7:**
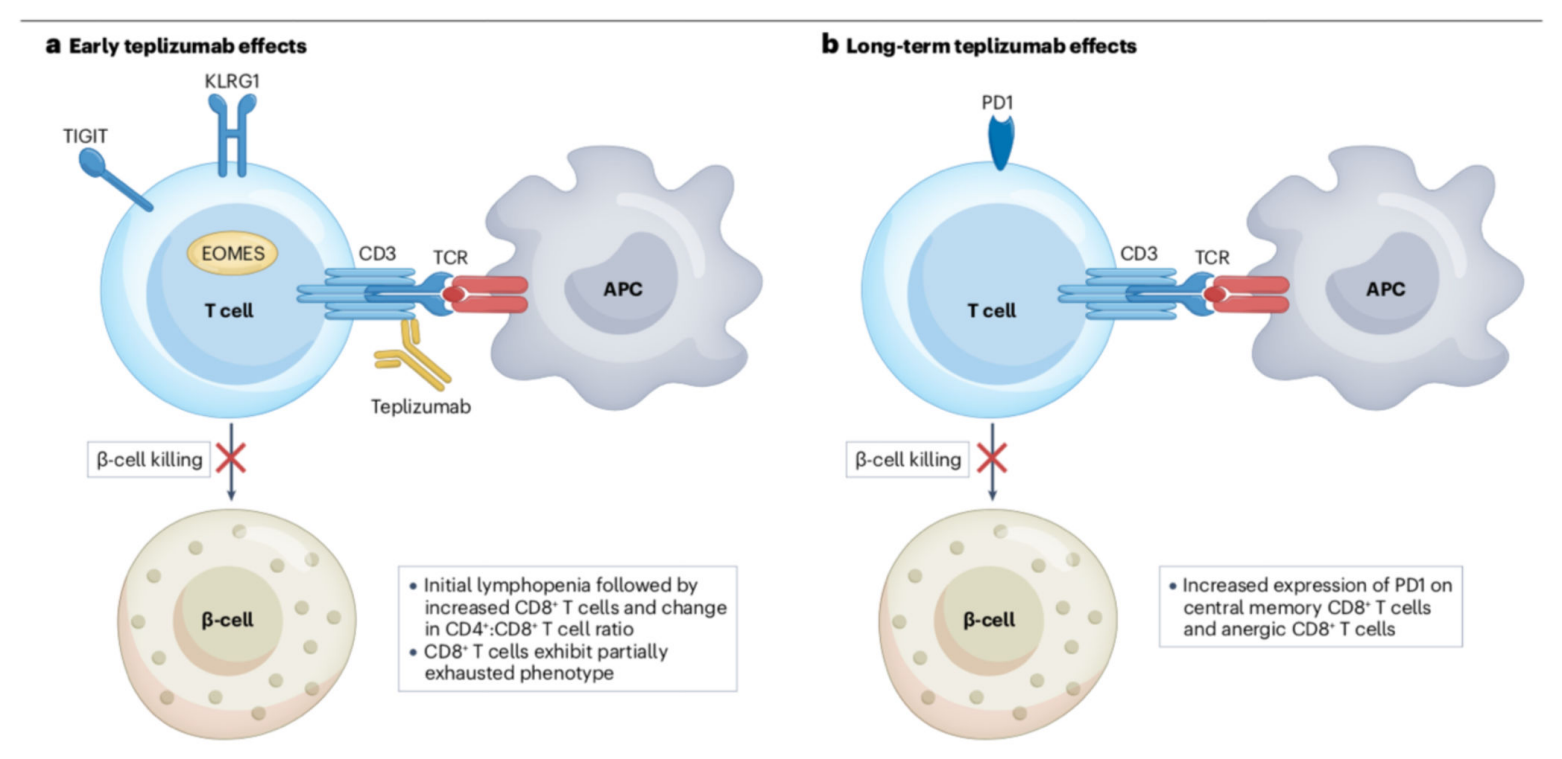
Mechanism of action of the CD3-targeting monoclonal antibody teplizumab. Early studies in animal models of the Fc receptor (FcR) non-binding monoclonal antibody (mAb) teplizumab have shown that by binding CD3, it induces a partial T cell receptor signal that induces clonal anergy and, thereby, inhibits β-cell killing^233^. In vitro and in vivo experiments have demonstrated that it induces a relative expansion of CD8^+^ T cells and the secretion of IL-10; similar results were also found with a FcR non-binding version of the CD3-targeted mAb. Moreover, teplizumab induced a transient decline in circulating lymphocytes, which reflected the migration of T cells to the gut wall, wherein activation signals led to the induction of CD4^+^TGFβ^+^ or CD8^+^IL-10^+^ T cells^234,235^. Moreover, FcR non-binding CD3-targeted mAbs induced a selective preservation of Treg cells, and another study has reported that teplizumab induced TGF β^+^ Treg cells that have a role in the restoration of self-tolerance in the pancreatic draining lymph nodes^236,237^. **a**, Observations from the initial clinical trials showed activation of CD8^+^ T cells in vivo, and a transcriptome analysis of bulk RNA from peripheral blood mononuclear cells (PBMCs) from clinical responders in the AbATE trial, showed changes in CD8^+^ T cells that were later found to involve the induction of the transcription factor EOMES and increased expression of the receptors KLRG1 and TIGIT, suggesting that the cells were ‘partially exhausted’^197,198,238.^ **b**, A long-term follow-up study of responders from the AbATE trial has shown an increased expression of PD1 on CD8^+^ T cells and an increase in PD1^+^ central memory CD8^+^ T cells and anergic CD8^+^ T cells, which may explain the long-term effects of teplizumab^239^. Autoantigen-reactive T cell receptors from CD8^+^ T cells have weak avidity for the antigen–MHC complex. The partial agonist signal delivered by the FcR non-binding CD3-targeted mAb may preferentially affect TCR signalling in response to such weak interactions, given that responses to conventional antigens such as viral antigens recover quickly after drug administration. APC, antigen-presenting cell.

The TN10 study, conducted by the National Institute of Diabetes Digestive and Kidney Diseases T1D TrialNet, tested whether a single course of teplizumab would delay or prevent progression to clinical disease in individuals with pre-diabetes, defined as stage 2 T1D with a high-risk progression to stage 3 T1D. The median time from enrolment to diagnosis with stage 3 T1D was 27 months in placebo-treated patients, which was delayed by approximately 2 years with teplizumab treatment^3,204^. However, some participants were not diagnosed with T1D for much longer periods of time (that is, >10 years after treatment). In this study, stimulated C-peptide responses, which were lower in this at-risk cohort than in healthy age-matched individuals, improved rapidly after treatment^3,205^. The mechanism of action of teplizumab is attributable to the nature of the partial agonist signal that is delivered to T cells (Fig. 7). These findings, based on studies of peripheral blood cells from treated patients, extend the observational studies that slow rates of spontaneous disease in at-risk individuals correlate with T cell exhaustion signatures identified in treated patients by expression of an EOMES signature and the expression of TIGIT and KLRG1 on CD8^+^ T cells^206^.

### Augmenting immune regulatory function

To enhance the number and function of T_reg_ cells, a clinical trial was initiated to test a combination of sub-cutaneous administration of IL-2 (4.5 × 10^6^ IU three times per week for 1 month) and rapamycin. However, it showed a deterioration of C-peptide responses along with expansion of NK cells, CD8^+^ effector cells and eosinophils^207^. Other groups have carefully titrated the dose of recombinant human IL-2 (rhIL-2) in order to selectively enhance the expansion of T_reg_ cells without expanding NK cells or CD8^+^ effector T cells^208,209^. In a phase I–II trial, a low dose of rhIL-2 was associated with C-peptide preservation for a small subgroup of study participants^210^. A more direct approach to harness T_reg_ cells involves cellular therapy, which was enabled by technological advances in expanding T_reg_ cells ex vivo^211^. In a pilot trial, T_reg_ cell adoptive cellular therapy was given alone or with rhIL-2 to enhance T_reg_ cell survival. The infusions were considered safe but the combination trial with rhIL-2 was stopped because of transient worsening of C-peptide responses. Mechanistic studies have shown expansion of CD8^+^ effector T cells in patients receiving rhIL-2 (refs. 212,213). However, a subsequent phase III trial of expanded autologous T_reg_ cells failed to meet its clinical end point of improvement in stimulated C-peptide responses at 1 year^212,213^. Redirecting T cell specificity through TCR gene transfer or by using chimeric antigen receptor expression may be required to improve the efficacy and safety of this approach. Moreover, strategies to improve the survival and stability of transferred cells are also being developed^214^.

## Conclusions and future directions

Decades of mechanistic research are beginning to bear fruit in the quest to modulate the autoimmune response to pancreatic β-cells in T1D, as highlighted by the approval of teplizumab. Progress has been made in understanding the differentiation state of pathological CD4^+^ and CD8^+^ T cells and the nature of the autoantigens that drive the disease. More remains to be learned about the mechanisms that underpin stage 1 and stage 2 T1D and the molecular pathways and environmental influences that promote disease progression. We anticipate that the application of high-parameter imaging techniques to pancreatic tissue samples from patients or mice with T1D will allow new insights into the identity and phenotype of islet-infiltrating immune cell populations. The results of clinical studies have highlighted several areas that could be further developed.

Importantly, trials to date have targeted single molecules or cell types. The failure of current therapies to provide long-term protection suggests that combinations or repeated administration of therapeutic agents are needed. For example, targeting B cells, T_FH_ cells and IL-21 production simultaneously may prevent the activation of pathogenic effector CD8^+^ T more efficiently than targeting single components, but such an approach has not yet been trialled^215^. Mechanistic studies of samples from individuals with T1D who fail to respond to immune-targeted drugs, or in whom the efficacy of successful drugs wanes, have suggested rational combinations of drugs to prevent disease recurrence — for example, the T cell signature associated with poor response to rituximab (see in the previous section) has led to a clinical trial in which abatacept is given after rituximab. The recent success of the JAK inhibitor baricitinib in patients with recent-onset T1D may involve the inhibition of inflammatory pathways involved in immune cell activation, but it may also prevent the damaging effects of inflammatory cytokines directly on β-cells^217^. Agents to improve β-cell function, expand their mass or stabilize glycaemia, when combined with an immune therapy, may achieve the desired metabolic control that occurs only with functioning endogenous β-cells. A combination of an agent to arrest the destructive immune response that occurs at or prior to diagnosis, followed by a more specific treatment that can be administered repeatedly, such as an antigen that is delivered in a tolerogenic manner, may represent a safe approach to sustain endogenous β-cell function. Therapies with smaller target cell populations, including antigen-specific therapies, may allow repeated administration with acceptable safety profiles.

Not all therapies have or would be expected to work in all patients: genetic, demographic and immune markers may be developed to optimize the choice of drugs for individuals. In addition, the timing of interventions may be important. Different therapies may work maximally at different disease stages, and delayed intervention may present the added challenge of memory responses. It is important to consider that even in patients at stage 1 T1D, an auto-immune response has already been initiated. Primary prevention is clearly an important goal; however, testing such approaches would require very large enrolment, a very safe agent and long follow-up. Nonetheless, with the approval of the first immune-cell targeted therapy to change the course of T1D, a new frontier has been opened.

## Figures and Tables

**Table 1:** Measures of autoimmunity and disease activity in individuals with and at risk for T1D.

Antigen, measured parameter or technique	Comment	Challenges
**Autoantibody assays**		
Unknown	Is let cell antibody (ICA) was the first auto antibody described; involves identification of serum binding to group O pancreas; not specific for β-celIs	The levels of autoantibodies may fluctuate and they do not reflect active β-cell killing
Glutamic acid decarboxylase 65 (GAD65)	GAD65 is an enzyme that synthesizes the neurotransmitter GABA at the nerve terminalsand synapses. It is found in islets of Langerhans. The affinity of autoantibodies to this antigen is higher in individuals who carry HLA-DR3. The presence of high affinity glutamic acid decarboxylase antibody (GADA) may indicate high risk^218^	
ICA512/IA-2(IA2A)	An autoantigen with a tyrosme phosphatase-like domain. Children who seroconverted early in life (median age >2years) and develop insuIin autoantibodies(IAA) and insulinoma-associated antigen 2 autoantibodies (IA2A) have the highest risk of progression to T1D, and this risk was unaffected by the presence or absence of GADA status^219^	
ZnT8(ZnT8A)	Zinc transporter found on β-cells	
(pro)lnsulin(lAA)	Antibodies to insulin and pro insulin, which are predominantly found in children carrying HLA-DR4. The presence of lAA to(pro) insulin allows to identify high risk of progression toTID^220–222^	
Modified (cit rullinated) antigens	Antibodies toc itrullinated glucokinase and carbonylated P4Hb^146,223^. These can be found with T cells reactive to modified peptides	
**β-Cell a stays**		
Measurement of *INS* DNA with unmethylated CpG sites	This assay detects epigenetic marks of β-cell-derived *INS* DNA in the serum	The half life of circulating DNA is short and the number of dying β-cells at any one time is small
Relative pro insulin levels	Reduced pro insulin processing and increased relative levels of pro insulin (compared to C-peptide or insulin), as measured by enzyme linked immunosorbent assays (ELISA) in serum, are associated with β-cell stress responses	Choice of assay may affect the sensitivity of the assay. Age is a confounder
Functional assays	Impaired first-phase insulin secretory responses and glucose intolerance are indicators of β-cell dysfunction	In prevention studies, there is a poor correlation between C-peptide responses and glucose responses to an oral glucose tolerance test (OGTT)^3^. Assessment of msulin secretory dynamics are not widely used and their sensitnaty to successful immune therapies is not clear.
**B cell assays**		
Anergic B cells	At-risk patients appear to have lower levels of anergic B cells^82^	B cells are not the direct cause of β-cell killing but may facilitate au tore active T cell responses by acting as antigen-presenting cells. The frequency of anergic B cells has not been evaluated as a biomarker
**T cell assays**		
EL Is pots/flow cytometry cell activation	CD4* T cells that are specific for multiple autoantigens can be detected^224,225^	The sensitrvity of the assay is limiting because the frequency of autoantigen-specific cells is on the order of 1:10^5^ or less
Tetramers and Qdot probes with ’fluorescent’ labels	Class land class II tetramers have been used to identify autoantigen-specific T cells from enriched cel (populations. The specificity for identifying anti gen-specific Toe Ils may be improved by using multiple fluorochromes for tetramers or Odots^226^. Preproinsulin-reactive cells have been linked to β-cell killing^110^	The frequency of autoantigen-specific T cells in the peripheral blood may be similar in individuals with T1D and healthy controls. Therefore, the frequency of the autoantigen-specific T cells may not be a specific biomarker of the disease
T cell libraries	CD8* T cell libraries detect auto anti gen-specific T cells from expanded enriched CD8* Tcell subpopulations^227^	Only the relative frequency of the autoantigen-specific cells can be deter mined
Proliferation assays	Proliferatrve or activation responses (for example, expression of C (MOL) to pools or single peptides can be measured ^228^	The absolute frequency of cells can not be ascertained. Cultures are frequently supplemented with cytokines which may increase background responses
Immunoblot	Is let antigens transferred to nitrocellulose and added to cell proliferation assays ^225^	The precise antigen(s) are not defined.

This table lists assays that have been used clinically to identity individuals with and at risk for type 1 diabetes (T1D) and to monitor disease activity.

## Box 1 Road to development of therapies for the treatment and prevention of T1D

- **Discovery Stage:** Concept development based on cellular mechanisms, genetics and tissue pathology. Targets for therapy, timing of therapy and adverse events are investigated. Preclinical models allow to study genetics and cellular mechanisms, as well as testing therapeutics for efficacy, timing and adverse events. NOD mice, transgenic NOD mice (such as BDC2.5), RIP LCMV transgenic mice and DO11 × RIP-mOVA mice are typically used. Moreover, human and mouse islets can be studied in vitro and ex vivo. For example, nPOD has provided pancreatic specimens from patients at-risk and those who have died with T1D (ref. 240).
- **Early-phase clinical studies:** These studies of limited size aim to determine the safety, biological and clinical efficacy, proof of mechanism and early evidence for ‘prospect of benefit’. They are most often carried out in adults but may include children (healthy individuals or those with recent-onset stage 3 T1D). They can involve testing new drugs or repurposing of approved drugs such as abatacept and rituximab173,179.
- **Later-phase clinical studies:** These studies test the preservation of β-cell function and clinical parameters and the diagnosis of stage 2 or 3 T1D. Individuals are typically diagnosed with new-onset T1D (around 100 days from diagnosis) and can include children. They also include smaller-size prevention studies in individuals with stage 1 or 2 T1D.
